# Echocardiographic evaluation of myocardial dysfunction in term neonates with perinatal asphyxia

**DOI:** 10.12669/pjms.40.9.9501

**Published:** 2024-10

**Authors:** Javaria Rasheed, Muhammad Khalid, Iram Nawaz, Barera Maryam

**Affiliations:** 1 Javaria Rasheed, FCPS, Department of Pediatric Medicine, Nishtar Medical University Hospital, Multan, Pakistan; 2 M. Khalid, FCPS, MSc (Epidemiology & Biostatistics), Department of Pediatric Medicine, Nishtar Medical University Hospital, Multan, Pakistan; 3 Iram Nawaz, FCPS Department of Pediatric Medicine, Bakhtawar Amin Medical & Dental College, Multan, Pakistan; 4 Barera Maryam, MBBS, Department of Pediatric Medicine, Nishtar Medical University Hospital, Multan, Pakistan

**Keywords:** Asphyxia neonatorum, Echocardiography, Pulmonary hypertension, Neonates, Cardiac dysfunction

## Abstract

**Background & Objective:**

One third of the neonatal deaths worldwide are attributed to perinatal asphyxia. We aimed to determine the prevalence and determinants of cardiac dysfunction, through echocardiographic evaluation, in term neonates with perinatal asphyxia.

**Methods:**

This cross-sectional study was conducted at a tertiary care setting over a period of six months from 1^st^ January 2021 to 30^th^ June 2021. Term neonates, weighing ≥ 2500 grams, born with Apgar score < 7 and admitted within 48-hours of life were consecutively enrolled. Using Levene classification neonates were grouped into moderate and severe perinatal asphyxia. All neonates underwent transthoracic echocardiographic evaluation after 24-hours of stabilization and within 72-hours of life. Descriptive statistics are calculated, and logistic regression analysis is done to determine the risk factors of myocardial dysfunction.

**Results:**

Among 166 neonates, 53% (n=88) were males, mean gestational age was 38.1 ± 0.89 weeks. Moderate asphyxia was present in 121 (72.9%). Most common echocardiographic finding was pulmonary hypertension in 50% followed by patent ductus arteriosus (PDA) in 37.2% and mitral regurgitation in 6.6%. Myocardial dysfunction was detected in 28.9% of the neonates. Three independent determinants of myocardial dysfunction were severe asphyxia [adjusted odds ratio (aOR) 5.01, 95% CI 2.2-11.4; p-value <0.001], having patent ductus arteriosus (aOR 5.11, 95% CI 2.2-11.8; p-value < 0.001) and delivery through cesarean section (aOR 2.65, 95% CI 1.2-5.9; p-value 0.02).

**Conclusions:**

Myocardial dysfunction among neonates with perinatal asphyxia is common and severity of asphyxia, mode of delivery and presence of patent ductus arteriosus are important determinants.

## INTRODUCTION

Perinatal asphyxia is estimated to affect 2-10/1000 term newborns worldwide. The World Health Organization reports four million deaths of neonates annually occurring due to perinatal asphyxia and the 3^rd^ most frequent cause of neonatal deaths after prematurity and infections. In developed countries incidence of neonatal deaths due to perinatal asphyxia is < 0.1% but in developing countries it ranges from 4.6/1000 to 7–26/1000 live births.^1^ Perinatal asphyxia causes multi organ failure which includes neonatal encephalopathy.^2^ Cardiovascular compromise and organ dysfunctions occur in cases of hypoxic ischemic encephalopathy (HIE), especially if it is related with fetal acidosis of pH <7 and APGAR (Appearance, Pulse, Grimace, Activity, Respiratory) score of 0-3 at 5th minute after birth.^3^

Brain, heart, respiratory tract, kidneys, intestine, and bone marrow are target organs involved in perinatal asphyxia.^4^ The most common abnormalities involve kidneys (50%) followed by neurological system (28%), heart (25%), and lungs (23%).^5^ During severe hypoxia cardio-vascular changes may occur which include tricuspid valve incompetence, mitral valve insufficiency, pulmonary hypertension and transient myocardial ischemia. These disturbances ultimately cause cardiac stunning, poor contractility and hypotension.^6^

In developing regions, perinatal asphyxia constitutes one of the common causes of deaths in newborns. It may cause multiple organ failure including heart.^7^ Cardiovascular impairment could be easily missed as more focus is given to other end organ disturbances. This study aimed to determine the prevalence and determinants of cardiac dysfunction using echocardiography in term asphyxiated neonates of our local population thus providing evidence for treating physicians to be more vigilant in diagnosing and promptly managing cardiac dysfunctions in perinatal asphyxia.

## METHODS

This cross-sectional study at the neonatal unit of the Pediatric Department of Nishtar University Hospital Multan commenced from January 1, 2021, to June 30, 2021, after receiving approval from the institutional Ethical Review Committee (23483/NM&H dated December 3, 2020). Neonates meeting criteria (≥ 37 weeks gestation, weight ≥ 2500 grams, delayed cry at birth, Apgar score ≤ 6 at 5 minutes, admitted within 48 hours) were consecutively enrolled with parental informed consent. Exclusions encompassed congenital heart diseases (except PDA), major anomalies, and neonatal sepsis. Perinatal asphyxia was defined as delayed cry, necessitating resuscitation, with Apgar score < 7 at five minutes.^8^ Neonates were categorized by Levene classification into moderate and severe perinatal asphyxia.^9^

Newborns underwent echocardiography 24 hours post-birth, conducted by a Pediatric cardiologist using a Toshiba 2D Nemo XG Echocardiography machine with a 10 MHz transducer. Left ventricular function was assessed through parameters such as left ventricular ejection fraction (LVEF), fractional shortening (FS), left ventricular end-diastolic diameter (LVEDD), and left ventricular end-systolic diameter (LVESD). Additional cardiac assessments included tricuspid regurgitation (TR), mitral regurgitation (MR), and the presence/severity of pulmonary hypertension. Functional pulmonary hypertension was defined as TR between 25-40 mm Hg within 72 hours of birth. Myocardial dysfunction was indicated by LVEF <55%, FS <26%, and pulmonary artery hypertension with TR and/or MR. Newborns were uniformly managed according to the hospital protocol, but selective/total body hypothermia was unavailable at the institutional level. The sample size of 166 neonates was determined, assuming 30% systolic dysfunction based on Shahidi M et al.^10^ report, with a 95% confidence level and 7% absolute precision. Statistical analyses, using SPSS version 25, included descriptive statistics in mean (±SD) or median (IQR), and frequencies/percentages. Logistic regression assessed myocardial dysfunction determinants. Factors with p-value ≤ 0.20 at the bivariate level entered the multivariable model, and p-value < 0.05 was deemed significant. Multicollinearity, assessed with variance inflation factor (VIF), used a cut-off value of 5. Results present crude and adjusted odds ratios with 95% confidence intervals.

## RESULTS

The study enrolled 166 neonates, comprising 121 (72.9%) with moderate and 45 (27.1%) with severe perinatal asphyxia. Myocardial dysfunction was identified in 28.9% (n=48) of neonates. Median postnatal inclusion age was 39 (IQR - 12) hours. Mean gestational age was 38.1±0.89 weeks, with 53% (n=88) being males. Average birth weight was 2.97±0.42 kilograms, and 54.8% (n=91) were delivered via cesarean section. Intravenous magnesium sulfate was used in 35.5% (n=59), and inotropes in 19.3% (n=32) of asphyxiated neonates. Enrollment age was significantly higher in moderate (41, 10 vs. 36, 14, p-value 0.007) versus severe asphyxia. Neonates with severe asphyxia had a higher gestational age (38.33±0.91 vs. 37.99±0.87, p-value 0.03). A significantly higher proportion of moderate asphyxia neonates did not require inotropic support compared to severe asphyxia (77.6% vs. 22.4%, p-value 0.005) (Table-I).

**Table-I:** Characteristics of neonates admitted due to perinatal asphyxia (N=166).

Characteristics	Overall (N=166)	Myocardial Dysfunction (n=48)	No Myocardial Dysfunction (n=118)	P value
Age (hours)*	39 (12)	38 (11)	41 (13)	0.13^‡^
Gestational age(weeks) ^†^	38.1±0.89	38.1 ± 0.91	38.1 ± 0.88	0.86^§^
***Gender***				
Male	88 (53)	29 (33.0)	59 (67.0)	0.22^||^
Female	78 (47)	19 (24.4)	59 (75.6)	
Weight^†^	2.97±0.42	2.98 ± 0.41	2.97 ± 0.43	0.98^§^
***Mode of delivery***				
Spontaneous Vaginal	75 (45.2)	14 (18.7)	61 (81.3)	0.008^||^
Cesarean section	91 (54.8)	34 (37.4)	57 (62.6)
***Severity of Perinatal Asphyxia***				
Moderate	121 (72.9)	25 (20.7)	96 (79.3)	< 0.001
Severe	45 (27.1)	23 (51.1)	22 (48.9)
***Use of Magnesium Sulphate (MgSO_4_)***				
Yes	59 (35.5)	17 (28.8)	42 (71.2)	0.98^||^
No	107(64.5)	31 (29)	76 (71.0)
***Use of inotropes***				
Yes	32 (19.3)	13 (40.6)	19 (59.4)	0.104^||^
No	134 (80.7)	35 (26.1)	99 (73.9)	

* median (IQR), † = mean (±standard deviation), ‡ = Mann-Whitney U test, § = Independent sample t-test, || = Pearson chi-square test.

The study revealed a mean left ventricular ejection fraction (%) of 62.5±11.3 and fractional shortening (%) of 31.5±7.0. Left ventricular end-diastolic and systolic diameters (mm) averaged 14.9±2.5 and 7.5±3.2, respectively. Mean tricuspid regurgitation was 36.7±13.4 mm of Hg. Mitral regurgitation was present in 6.6% (n=11), pulmonary hypertension in 50% (n=83), and PDA in 37.2% (n=45) of neonates. The jet of tricuspid regurgitation was stronger in neonates with myocardial dysfunction compared to those without (45.79 ± 11.2 vs. 32.93 ± 12.5, p-value < 0.001). Pulmonary hypertension was significantly more frequent in neonates with myocardial dysfunction (57.8% vs. 42.2%). Neonates without myocardial dysfunction exhibited only mild pulmonary hypertension, while moderate and severe cases were exclusive to those with myocardial dysfunction (Table-II).

**Table-II:** Echocardiographic findings in neonates admitted due to perinatal asphyxia (N=166).

Characteristics	Overall (N=166)	Myocardial Dysfunction (n=48)	No Myocardial Dysfunction (n=118)	P value
LVEF (%)*	62.5±11.3	46.7 ± 5.5	68.97 ± 4.8	<0.001†
FS (%)^α^	31.5±7.0	22.6 ± 3.7	35.13 ± 4.3	<0.001†
LVEDD (mm)*	14.9±2.5	15.33 ± 2.4	14.73 ± 2.5	0.16†
LVESD (mm)*	7.5±3.2	7.73 ± 3.0	7.44 ± 3.3	0.60†
TR (mm of Hg)*	36.7±13.4	45.79 ± 11.2	32.93 ± 12.5	< 0.001†
***Mitral Regurgitation***				
Yes	11 (6.6)	5 (45.5)	6 (54.5)	0.21‡
No	155 (93.4)	43 (27.7)	112 (72.3)
***Pulmonary Hypertension***				
Yes	83 (50)	48 (57.8)	35 (42.2)	< 0.001‡
No	83 (50)	0 (0.0)	83 (100)
***Severity of Pulmonary Hypertension (n=83)***				
Mild	35 (21.1)	0 (0.0)	35 (100)	< 0.001‡
Moderate	31 (18.7)	31 (100)	0 (0.0)
Severe	17 (10.2)	17 (100)	0 (0.0)
***Patent Ductus Arteriosus***				
Yes	45 (37.2)	22 (48.9)	23 (51.1)	0.001‡
No	121(62.8)	26 (21.5)	95 (78.5)

LVEF: Left ventricular ejection fraction, FS: Fractional shortening, LVEDD: Left ventricular end diastolic diameter, LVESD: Left ventricular end systolic diameter, TR: Tricuspid regurgitation

* mean (± SD), † Independent sample t-test, ‡ Pearson chi-square test / Fischer’s exact test.

Three independent factors predicting the development of myocardial dysfunction were identified. Neonates with myocardial dysfunction had higher odds of suffering from severe asphyxia [adjusted odds ratio (aOR) 5.01, 95% CI 2.2-11.4; p-value< 0.001], having patent ductus arteriosus (aOR 5.11, 95% CI 2.2-11.8; p-value < 0.001) and delivery through cesarean section (aOR 2.65, 95% CI 1.2-5.9; p-value 0.02). The Hosmer–Lemeshow test indicated that model is well fitted (p-value 0.77). Model fitness was also evident through classification table (76.5% correctly classified) and the receiver operating characteristics (ROC) curve with an area under curve of 0.78 (Table-III).

**Table-III:** Determinants of myocardial dysfunction in neonates admitted due to perinatal asphyxia (N=166).

Determinant	Un-adjusted OR (95% CI)	P value	Adjusted OR (95% CI)	P value
**Age (hours)**	0.98 (0.94-1.02)	0.37	-	-
***Gender***		0.22	-	-
Female	1			
Male	1.5			
***Gestational Age***		0.66	-	-
37-38 weeks	1			
39-40 weeks	0.85			
***Birth weight***		0.93	-	-
Up to 3.0 kg	1			
> 3.0 kg	1.03			
***Mode of delivery***		0.009	2.65 (1.2-5.9)	0.02
Normal	1			
Cesarean section	2.60			
***Grade of Asphyxia***		< 0.001	5.01 (2.2-11.4)	< 0.001
Moderate	1			
Severe	4.01			
***Use of MgSO_4_***		0.98	-	-
No	1			
Yes	0.99			
***Use of Inotropes***		0.11	-	-
No	1			
Yes	1.94			
***Presence of PDA***		0.001	5.11 (2.2-11.8)	<0.001
No	1			
Yes	3.49			

No multicollinearity and no interaction. Hosmer Lemeshow test, P value=0.77. Classification table 76.5% correctly classified. ROC (Area under the curve) is 0.78.

## DISCUSSION

In our study, myocardial dysfunction was observed in 28.9% of neonates. Severe asphyxia, presence of PDA, and cesarean delivery were identified as determining factors. Tanna K et al^11^ reported a higher prevalence of 67.5%, potentially attributed to 75% of their neonates experiencing severe asphyxia, compared to 27% in our study. Consistent with our findings, severe asphyxia emerged as a major determinant of myocardial dysfunction.

Half of the neonates developed pulmonary hypertension, exclusively seen in those with myocardial dysfunction, including all cases of moderate and severe pulmonary hypertension. Sayeed MA et al in their study corroborated these results, identifying pulmonary hypertension, tricuspid regurgitation, and cardiac dilatations as common abnormalities.^12^ In neonates, pulmonary hypertension induces remodeling, impacting cardiolipin biosynthesis and remodeling enzymes, ultimately leading to both right and left ventricular dysfunction.^13^,^14^

Severity of cardiac impairment is determined by the degree of perinatal asphyxia.^15^ The transitory myocardial ischemia may manifest in the mild form as tachypnea or as severe as cardiogenic shock.^16^ Myocardial insult or ischemic myocardial necrosis in both ventricles occur due to severe asphyxia and poor perfusion.^3^ Also, reperfusion injury may produce severe cardiovascular disturbances with high morbidity and mortality through degradation of cardiac myosin light chain 1 protein (MLC1) by matrix metalloproteinase-2 (MMP-2).^17^

Our study did not reveal diastolic dysfunction; however, Shahidi M et al, demonstrated >50% occurrence in newborns with moderate or severe asphyxia, noting more severity in severe cases. Systolic functions were statistically influenced by perinatal asphyxia.^10^ This might be attributed to our institutional protocol, which restricted intravenous fluids and inotropic support for perinatal asphyxia management, potentially masking diastolic dysfunction. Another study observed higher LVED and LVES diameters in asphyxiated newborns, while fractional shortening and ejection fraction were reduced.^18^

Our study revealed an increasing trend of tricuspid regurgitation (TR) and pulmonary hypertension in neonates with myocardial dysfunction compared to those without. Bhasin H et al reported significant echocardiographic parameter discrepancies with increasing hypoxic ischemic encephalopathy (HIE) severity (p-value <0.05).^19^ Patent ductus arteriosus (PDA) was frequently observed post-perinatal asphyxia, aligning with our findings, while Shahidi M et al noted PDA in 62% of asphyxiated neonates.^10^ Mechanism involved in PDA development has been explored in asphyxiated lambs.^20^ In our study, moderate to large-sized PDAs could contribute to cardiac dysfunction and pulmonary hypertension.^21^

The prevalence of pulmonary hypertension in our study aligned with an Indian study (43.9%).^22^ In our cardiac dysfunction group, the tricuspid regurgitation (TR) jet was stronger compared to the non-cardiac dysfunction group. Jain D et al identified TR as the most common valvular lesion (35%), followed by mitral regurgitation (22%) in asphyxiated neonates.^23^ Another study reported a 23% incidence of TR.^24^ Variations in TR and mitral regurgitation incidences may be linked to patient selection, coexisting pulmonary hypertension, and echocardiography timing. In asphyxiated newborns, TR could result from papillary muscle ischemia, akin to coronary artery disease mechanisms in adults.^25^

### Limitations:

Major strengths include a large sample size and early evaluation by a consultant pediatric cardiologist. However, being a single-center study and some neonates on prior inotropic support are limitations, potentially masking actual cardiac dysfunction prevalence.

## CONCLUSION

Perinatal asphyxia often impairs myocardial functions more than anticipated. Timely echocardiography aids accurate evaluation, potentially reducing mortality and morbidity.

### Authors Contribution:

**JR:** Designed the study, acquired data and responsible for accuracy or integrity of the study.

**MK:** Analyzed the data, interpreted results and 1^st^ manuscript draft.

**IN:** Literature review, data acquisition, critical review of manuscript.

**BM:** Data acquisition, manuscript writing and critical review.
